# The character strengths of class clowns

**DOI:** 10.3389/fpsyg.2014.01075

**Published:** 2014-09-30

**Authors:** Willibald Ruch, Tracey Platt, Jennifer Hofmann

**Affiliations:** Personality and Assessment, Institute of Psychology, University of ZurichZurich, Switzerland

**Keywords:** class clown, character strengths, VIA-Youth, signature strengths, life satisfaction, positive psychology

## Abstract

Class clowns traditionally were studied as a type concept and identified via sociometric procedures. In the present study a variable-centered approach was favored and class clown behaviors were studied in the context of character strengths, orientations to happiness and satisfaction with life. A sample of 672 Swiss children and adolescents filled in an 18 item self-report instrument depicting class clown behaviors. A hierarchical model of class clown behaviors was developed distinguishing a general factor and the four positively correlated dimensions of “identified as a class clown,” “comic talent,” “disruptive rule-breaker,” and “subversive joker.” Analysis of the general factor showed that class clowns were primarily male, and tended to be seen as class clowns by the teacher. Analyses of the 24 character strengths of the VIA-Youth (Park and Peterson, 2006) showed that class clowns were high in humor and leadership, and low in strengths like prudence, self-regulation, modesty, honesty, fairness, perseverance, and love of learning. An inspection of signature strengths revealed that 75% of class clowns had humor as a signature strength. Furthermore, class clown behaviors were generally shown by students indulging in a life of pleasure, but low life of engagement. The four dimensions yielded different character strengths profiles. While all dimensions of class clowns behaviors were low in temperance strengths, the factors “identified as the class clown” and “comic talent” were correlated with leadership strengths and the two negative factors (“disruptive rule-breaker,” “subversive joker”) were low in other directed strengths. The disruptive rule breaking class clown was additionally low in intellectual strengths. While humor predicted life satisfaction, class clowning tended to go along with diminished satisfaction with life. It is concluded that different types of class clowns need to be kept apart and need different attention by teachers.

## Introduction

Most classrooms have a few students who joke a lot and who make others in the room laugh. These are commonly called “class clowns.” Students that take on this role may disrupt class with their jokes and wisecracks, may make silly noises or pull weird faces, bump into imaginary walls, copy the teacher behind their back, and may make wild comments that gets the whole class laughing uproariously. As other students may start imitating their behavior, this may get a class out of control and thus, the class clown may constitute a disciplinary problem for a teacher (Reed, 1989), even if it does not compare to more serious disciplinary problems (such as sexual or racial harassment, stealing or using abusive language).

Almost 40 years have elapsed since the classic study on class clowns by Damico and Purkey (1976, 1978) that first shed some light onto this common but overlooked phenomenon. Their study involved 96 class clowns (derived from a sample of 3500 eighth graders), mostly males (80 out of 96), that were compared to a randomly selected sample of 237 pupils. Analyses of teacher perceptions of students yielded that class clowns were significantly higher than non-clowns on asserting behaviors (i.e., speaking up and actively participating in class), attention seeking, unruliness, leadership, and cheerfulness, but lower in “accomplishing.” Class clowns themselves reported less positive attitudes toward the school authorities (i.e., teacher and principal) but there was no difference in their attitude toward classmates, the school in general, and the self. Finally, class clowns saw themselves as leaders and as being vocal in expressing ideas and opinions in front of their classmates. Damico and Purkey (1978) concluded that “… [a]dolescent clowns were found to have many behaviors and personal assessments in common with adult wits. They are male, leaders, popular, active, independent, creative, and have positive self-perceptions. Among adults, groups containing wits were found to possess higher morale, be more task-oriented, and better at solving problems than groups without wits (Smith and Goodchilds, 1959, 1963). Given the similarity between adolescent clowns and adult wits in other areas, it is safe to assume that clowns might make similar contributions to groups within schools” (p. 397).

These pioneering results were neither replicated nor refined or expanded—perhaps “… because of the difficulty of finding enough class clowns to make a meaningful analysis” (p. 186; Priest and Swain, 2002). Before continuing with this research it seem of interest to make a few adjustments. The first issue relates to the opposition of *the class clown as a type vs. class clowning behavior*. We propose that next to the person-centered type approach (i.e., identifying who is a class clown and who is not) a variable-centered approach (i.e., describing the behaviors that class clowns exhibit and study their dimensionality) should be pursued. The “type” approach suffers from several limitations. “Class clowns” (like the wit, or “organizational fool,” Kets de Vries, 1990) is a lay-concept, referring to an informal role (not a profession or a vocational entity like a circus or hospital clown) but this is not a scientific concept. As a type noun it emerged from everyday conversations about pupils and entered scientific discourse without further scrutiny. In the history of personality research types often disappeared once measurement started as one often found that there are no pure types (but gradual differences within the proposed types) and the behaviors associated to types turned out to be multidimensional. Then, the upper end of the dimension may be considered a “type” (i.e., the people above a cut-off value). Thus, for the present study descriptions of class clown behaviors were collected from the literature and entered in a list to be examined (Platt, 2012).

The second issue relates to methodology, namely the question of *use of sociometry vs. use of questionnaires*. We want to propose that next to the sociometric identification of class clowns (by teachers, classmates), also questionnaires (self-reports, peer-, and teacher-reports) are used to assess gradual differences among students on one global dimension (or several separate dimensions) of class clown behaviors. In the former case the number of nominations received matters, in the latter the quantification comes from the number of items that apply (i.e., the number of class clown behaviors that someone engages in). In both cases there is a variation and cut-off scores are used to eventually make a dichotomous judgment (i.e., class clown, no class clown). In the study by Damico and Purkey (1978) only those students that received 10 or more nominations by their peers were considered a class clown and those with more than 25 nominations were “super class clowns” (and assumed to be attention seekers). When the participants are asked (Priest and Swain, 2002) whether they consider themselves to be the class clowns the scores are already binary and need no further treatment. A questionnaire approach has not been pursued so far and it needs to accommodate several observations found from sociometric studies (using teachers and peers). For example, class clowns appear to be a minority in a class; most students are not class clowns and thus skewed distributions might be expected. The number of class clowns in the sample varied in previous studies and may be as low as 3% (Damico and Purkey, 1978) and as high as 21% (Priest and Swain, 2002). This is setting a benchmark against which the cut-off points will be tested.

The third issue relates to the search of characteristics of class clowns: *School behavior or general character strength?* We propose that not only classroom behaviors are used to predict who is nominated as a class clown, but also more general characteristics of a student, such as his or her character could be used to predict dimensions of class clown behaviors. The inclusion of the model of character strengths allows describing class clowns more specifically but also more comprehensively. In their model of the good character Peterson and Seligman (2004) first discovered six virtues to be found in many virtue catalogs across the globe and covering the last two millennia, namely wisdom and knowledge, courage, humanity, justice, temperance, and transcendence. In the next step they identified 24 character strengths (i.e., processes and mechanisms that lead to the virtues), namely appreciation of beauty, bravery, creativity, curiosity, fairness, forgiveness, gratitude, honesty, hope, humor, kindness, leadership, love, love of learning, modesty, open-mindedness, perseverance, perspective, prudence, religiousness, self-regulation, social intelligence, teamwork, and zest. These strengths are considered to be distinguishable routes to displaying one or more of the virtues. Furthermore, Peterson and Seligman (2004) postulate the existence of “signature” strengths, i.e., the strength that a person “… owns, celebrates, and frequently exercises” (p. 18). While strengths generally are defined to contribute to various fulfillments that constitute the good life, for the self and for others it is the signature strengths that are most fulfilling for a given individual. In fact the application of individual signature strengths were demonstrated to be related to positive life outcomes such as higher happiness and meaning and lower levels of depression (e.g., Seligman et al., 2005; Littman-Ovadia and Steger, 2010; Harzer and Ruch, 2012; Gander et al., 2013).

There are several reasons to study of dimensions of class clown behavior within a framework of the good character. First, despite the fact that class clowning has occasionally be seen as a disciplinary problem, class clowns will possess certain strengths. The study of Damico and Purkey (1978) found higher scores for the class clown not only for leadership but also for “cheerfulness”— this might be an indirect effect of the class clowns' comic talent, and suggests that the strength of humor might characterize class clowns, or even may be their signature strength. Second, the display of more destructive class clown behavior might be related to underdeveloped strengths thereby showing where interventions might be fruitful. It should be reminded that one of the criteria for character strengths is that a strength by one person does not diminish other people in the vicinity. Class clowning behaviors might be detrimental to others as teachers or students can be the target of a prank. Likewise, if strengths that keep students in flow; i.e., school-related strengths, are underdeveloped it might be that attention wanders off to other things and need for fun kicks in and students start entertaining themselves and others. Thus, strengths aimed at fostering healthy communities and at the acquisition of knowledge might be less developed. Third, strengths should be in balance and the combination of strengths might be a fruitful venue of study. Even if humor is a signature strength of a person, he or she will not necessarily engage in class clowning, for example if humor is balanced out by strengths of temperance. Finally, the use of the VIA-Youth not only allows studying the 24 individual strengths but also the five strength factors that have repeatedly been found, namely *leadership strengths* (i.e., leadership, humor, perspective, social intelligence, and bravery), *temperance strengths* (i.e., prudence, self-regulation, perseverance, open-mindedness, and honesty), *intellectual strengths* (i.e., curiosity, love of learning, beauty, and creativity), *transcendence strengths* (i.e., religiousness, zest, gratitude, love, and hope), and *other-directed strengths* (i.e., modesty, forgiveness, kindness, fairness, and teamwork). The simultaneous study of all 24 individual strengths and the five strengths factors will allow drawing a more differentiated picture of the strengths (or lack of strengths) of class clowns beyond the domains where hypotheses exist.

The present study aims at (a) a set of items that may be used to measure the level of involvement in class clown behavior that is higher for the group of identified class clowns and lower for non-class clowns. Next, (b) the study aims at investigating whether different dimensions of class clown behaviors can be distinguished. Furthermore, (c) the character strengths of class clowns will be examined (both for the global class clown dimension as well as the dimensions identified). Finally, (d) the relationship with orientations to happiness and global life satisfaction will be examined. Generally humor is a predictor of life satisfaction among adolescents (*r* = 0.32 in Ruch et al., 2014). However, a low fit of humor as signature strengths to classroom discipline might lead to frustration and diminished happiness with school, and eventually with life in general. There are different types of well-being. Peterson et al. (2005) distinguished among pleasure, engagement, and meaning, and while class clown pranks are conducive to pleasure they will be antagonistic to engagement.

Some hypotheses can be put forward. In class pupils have to suppress the need to act or say something unless it is their turn. This will also be the case for class clown behaviors, which often are not appropriate or wise to perform. Hence Hypothesis 1 states that class clown behaviors will correlate negatively with temperance strengths (e.g., prudence, self-regulation, endurance). Class clown behaviors will be shown for entertainment and fun and are meant to amuse. Hence Hypothesis 2 predicts that the life of pleasure, as well as humor will go along with class clown behavior. Next, based on Damico and Purkey (1978) Hypothesis 3 claims that leadership will be a predictor of class clown behavior, both as an individual strength and the leadership strengths factor (i.e., leadership, humor, perspective, social intelligence, and bravery). Hypothesis 4 states a negative relationship between the factor of other-directed strengths (i.e., modesty, forgiveness, kindness, fairness, and teamwork) and negative class-clown behaviors. Some pranks and class clown behaviors have a target, or a person that is not happy about the clowning. Hence one can postulate that that pronounced interpersonal strengths will make pupils refrain from destructive behaviors. Likewise, it is predicted that being high in strengths related to the acquisition of knowledge (Hypothesis 5) and pursuing a life of engagement (i.e., the tendency to experience flow; Hypothesis 6) will be less inclined to indulge in class clown behavior. Hypothesis 5 will be tested for the factor of intellectual strengths as well as those the individual school-related strengths, identified in prior studies on the basis of correlation with positive school functioning and overall school achievement of pupils, such as love of learning, perseverance, and prudence (Weber and Ruch, 2012; Weber et al., submitted). Furthermore, predictions can be made too regarding the signature strengths: humor may be expected to be a signature strength among class clowns (Hypothesis 7), however, humor as signature strengths may not lead to class clown behavior if prudence is high too (Hypothesis 8). Finally, life satisfaction is expected to correlate negatively with the negative class clowning behaviors (Hypothesis 9).

## Materials and methods

### Participants

The sample consisted of 672 German-speaking children and adolescents (40.7% boys; two did not indicate gender). Their mean age was 14.87 years (*SD* = 3.33; ranging from 10 to 18 years; 3 missings). Regarding the school type, 28.1% of the participants attended primary school, 29.9% attended secondary school, 19.5% attended a gymnasium/ high school, 10.9% were currently enrolled in an apprenticeship, and 10.7% indicated “other” (e.g., extra school year or gap year), 0.9% of the participants did not indicate their school level. Overall, 87% of the sample were Swiss citizens. Less than 1% of responses to individual items were missing, but df in analyses vary because of this rare missingness. We used listwise deletion to handle missing data in analyses with items, but when computing scales, we computed average values and ignored missing data as long as no more than four items were missing.

### Instruments

The *Class Clown Behavior Survey* (CCBS; Platt, 2012) is an 18 item self-report instrument assessing a variety of class clown behaviors in a 6-point answer format (1 = *totally disagree*, 2 = *largely disagree*, 3 = *partially disagree*, 4, *partially agree*, 5 = *largely agree*, 6 = *totally agree*). A total score is computed by averaging all items to indicate how strongly students display class clown behavior. Furthermore, two items (“My classmates would call me a class clown.” “In my class I am the class clown.”) were used in the class clown status index; i.e., to identify class clowns and separate them from non-class clowns. These two items correlated *r* = 0.80 with each other and only if a participant on average agreed to the statements (i.e., scores between 4 and 6) it was identified as a class clown.

*Class clown nomination* (teachers). Teachers of four classes comprising 80 pupils (46% boys; mean age 15.2 years) were provided lists of pupils and indicated independently from each other whom they considered to be a class clown. The nomination was done 10 month after the students had left the school. The number of nominating teachers varied between 5 and 7 depending on the class. Nine out of 80 students (i.e., 11.25%) were nominated at least once. Thus, there was agreement that 88.75% were not class clowns. No student was nominated by every teacher; the nomination rate was between 14.29 and 83.33%, with a mean of 45.19% (and the number of nominations varied between 1 and 5). This demonstrates that not all teachers see the student alike; either some raise a false alarm or others do not see the class clown behaviors, or the students only behave like a class clown with certain teachers. Some teachers might also be milder in their evaluation. The convergence with being a class clown in the self-rating is significant (*p* < 0.05) but does not exceed 0.35.

The *Values in Action Inventory of Strengths for Youth* (VIA-Youth; Park and Peterson, 2006) adapted to German by Ruch et al. (2014) consists of 198 items for the self-assessment of the 24 character strengths of the VIA classification (Peterson and Seligman, 2004). There are 7–9 items per character strength (about one third of the items are reverse coded) in a 5-point Likert-style format (from 1 = *not like me at all* to 5 = *very much like me*). The VIA-Youth proved to be reliable and valid (e.g., Park and Peterson, 2006; Ruch et al., 2014). The internal consistencies ranged from 0.67 (modesty) to 0.90 (religiousness) and yielded a median of α = 0.79 in this study. Factor scores for the factors of leadership, temperance, intellectual, transcendence, and other-directed strengths (cf. Ruch et al., 2014) were derived by a PCA with Oblimin rotation.

The *Orientation to Happiness measure* (OTH; Peterson et al., 2005) is an 18-item self-report questionnaire for the subjective assessment of life of pleasure, engagement, and meaning (six items each). It utilizes a 5-point Likert-scale (1 = *not like me at all* through 5 = *very much like me*). Cronbach alpha was 0.73, 0.71, and 0.77 in the present sample.

The *Students' Life Satisfaction Scale* (SLSS; Huebner, 1991) in a German version by Weber et al. (2013) is a seven-item measure for the self-assessment of global satisfaction with life utilizing a 6-point answer format (from 1 = *strongly disagree* to 6 = *strongly agree*). The SLSS total score is formed by averaging the seven items. Cronbach alpha was 0.85 in the present sample.

### Procedure

Data in this study were collected partly in schools (group testing during lessons conducted by instructed teachers), partly at the university (group testing of pupils with two female experimenters) and partly via the internet. All participants attended voluntarily, and all students provided the permission of their parents or legal guardians beforehand in writing or by clicking a control question at the beginning of the online assessment session. All participants firstly filled in the VIA-Youth and then proceeded to the measures assessing the class clown behaviors, satisfaction with life and the orientations to happiness. The sessions lasted around 2–3 h. In the group testing, breaks were initiated by the experimenter/teacher, in the individual sessions, the children and adolescents could take breaks when needed. None of the students was paid for participation. All students received written individualized feedback on their character strengths and additional information on the meaning of each of the character strengths of the VIA classification. The study complies with the requirements from the local research ethics committee basing on the APA standards. The authors confirm that they have reported all data exclusions. The sample size was estimated on the basis of prior studies that allowed to expect on average a 10% prevalence of class clowns.

### Data analysis

To investigate the CCBS two analyses were performed. First, a principal component analysis was performed on the inter-correlations of the 18 items of the CCBS to see whether all items load on the postulated general factor. Second, a hierarchical factor analysis (see Goldberg, 2006) was employed to see whether there is any meaningful structure to find beyond the first unrotated principal component (FUPC). Hierarchical factor analysis allows seeing how the factors unfold with increasing numbers of extracted factors (cf. Goldberg, 2006). In more detail, the first principal component was extracted and the factor scores were saved. Next, two factors were extracted, rotated according to the Oblimin criterion, and the factor scores were saved. This procedure was repeated for all solutions up to the fifth factor, which produced factors that did not have enough meaningful markers anymore. Solutions between one and five factors were examined in order to have the possibility to study the relations between factors of different stages of extraction. The factors were interpreted. Then, the factor scores of adjacent factor solutions were correlated with each other, and the salient relations (*r* > 0.45) were represented using arrows. This way, it can be shown how the factors unfold, i.e., how they split up or stay stable from solution to solution. The internal consistencies of the derived subscales of the CCBS questionnaire were estimated by the Cronbach's alpha coefficient. Correlations between strengths and class clown dimensions were controlled for age and gender.

## Results

### Prevalence of class clowns

Altogether, 85.7% of the participants disagreed to the statement “In my class I am the class clown” (“1 = absolutely disagree”: 51.7% “2 = largely disagree”: 21.9%, “3 = partly disagree”: 12.2%) and 14.3% agreed (“4 = partly agree”: 8.0%; “5 = largely agree”: 5.6%, “6 = absolutely agree”: 0.8%) to it. Thus, the ratings showed that the assignment to be a class clown was not a bimodal variable but continuous. Participants also infrequently indicated that others would think that they were the class clowns (“1”: 36.1%, “2”: 25.5%, “3”: 15.2%, “4”: 14.3%, “5”: 6.9%, “6”: 2.0%). There were 85 who answered affirmatively to both questions (i.e., that they were the class clowns and that others would think that they were a class clown). Using this class clown status index one can state that 12.7% of the participants in the samples considered themselves to be class clowns. Gender played a role (*p* < 0.01), as only 9.3% of the females declared that they were class clowns, but 17.6% of the boys did.

### Analyses of the class clown questionnaires

Cronbach's alpha was very high (α = 0.92). The class clown behavior was correlated with the class clown status index. A correlation of this index and the total score of the remaining 16 items yielded a coefficient of 0.66 (*p* < 0.001). Likewise, a *t*-test confirmed that the class clowns (*M* = 3.61; *SD* = 0.76) scored higher than the non-class clowns (*M* = 2.43; *SD* = 0.74), *t*_(665)_ = −13.49, *p* < 0.001, η^2^_*p*_ = 0.22. Thus, those who assign themselves to being a class clown also showed more class clown behavior, validating both measures. Therefore, an average score across all class clown items was computed and used in the subsequent analyses. Furthermore, for a subgroup of 73 students, teacher nominations of class clowns was available and it correlated with 0.35 (*p* < 0.05) with the class clown self-report.

*Identification of dimensions of class clown behaviors (or “types”).* While the inter-correlations among all items were positive (median = 0.38), the range (0.12 to 0.80) suggested that a structure may be uncovered. Therefore, the inter-correlations among the 18 items were subjected to a principal components analysis. Three eigenvalues exceeded unity (eigenvalues were: 7.70, 1.50, 1.40, 0.91, 0.79, and 0.74) with the first factor alone explaining 42.79% of the variance confirming that a single factor solution is possible. The scree-test suggested the retention of four factors, which explained 63.79% of the variance. Also, the hierarchical factor analysis showed a four-factor solution to be optimal, as the fifth only yielded two markers^1^. The different solutions between the first unrotated principal component (FUPC) and four Oblimin-rotated factors are displayed in Table 1.

**Table 1:** **Factor loadings of the 18 class clown items on a first unrotated principal component, and Oblimin rotated 2, 3, and 4 factor solutions**.

	**FUPC**	**O2.1**	**O2.2**	**O3.1**	**O3.2**	**O3.3**	**O4.1**	**O4.2**	**O4.3**	**O4.4**
04. My classmates would call me a class clown	0.73	0.60	0.21	0.79	0.26	−0.12	0.83	0.15	0.07	−0.04
09. In my class I am the class clown	0.72	0.70	0.08	0.84	0.14	−0.07	0.82	0.04	0.08	0.07
05. During class it does not take long until my humor draws all attention of all my classmates on me	0.73	0.55	0.28	0.69	0.32	−0.06	0.66	0.25	0.02	0.07
17. My classmates expect from me that I do silly things and make them laugh	0.74	0.80	−0.02	0.47	−0.01	0.46	0.42	−0.08	0.49	0.19
07. During class it does not take long, until something funny comes into my mind, that I can share with the person next to me	0.59	−0.05	0.84	−0.04	0.79	0.07	−0.03	0.83	0.02	−0.04
02. I quickly can think of funny things, that I could do in class	0.64	0.04	0.78	0.14	0.76	−0.02	0.12	0.79	−0.07	0.02
01. I have funnier ideas and sayings than the teacher	0.60	0.04	0.72	0.08	0.70	0.04	0.10	0.71	0.03	−0.03
10. When the teacher leaves the room, I make fun with the person sitting next to me	0.63	0.12	0.66	−0.09	0.61	0.34	−0.15	0.66	0.21	0.10
13. During class I say funny things to spread good mood	0.71	0.27	0.58	0.28	0.57	0.09	0.21	0.58	0.04	0.11
16. I don't share everything the teachers find important (grades, rules); on the contrary: I occasionally poke fun at them	0.60	0.49	0.17	−0.18	0.11	0.85	−0.09	0.05	0.87	−0.02
18. Some rules in class I find stupid and I laugh at them	0.61	0.50	0.18	−0.12	0.13	0.78	−0.02	0.06	0.83	−0.04
15. Even when the teacher says my humor is disruptive, most of the time I cannot stop it immediately and need to continue having fun	0.74	0.68	0.14	0.20	0.11	0.63	0.18	0.06	0.62	0.14
03. When the teacher says that it is very important, that we learn a lot, I am always the first that does not take that seriously	0.56	0.53	0.08	0.25	0.08	0.39	0.26	0.02	0.43	0.07
12. When the teacher turns away, I invent jokes that I write on paper to show it to my classmates	0.60	0.72	−0.10	0.35	−0.09	0.50	−0.15	0.05	0.05	0.84
11. During the breaks I play pranks on my classmates	0.66	0.78	−0.10	0.45	−0.09	0.46	−0.02	0.02	0.07	0.80
08. Only after have the attention of the entire class I stop joking about	0.51	0.74	−0.25	0.61	−0.21	0.21	0.28	−0.17	0.01	0.57
06. During the breaks my ideas for pranks are cleverer than those of the other kids	0.70	0.61	0.16	0.60	0.19	0.10	0.25	0.27	−0.16	0.57
14. I make the other kids laugh at what the teacher said or did	0.68	0.56	0.20	0.17	0.18	0.53	−0.06	0.23	0.30	0.43
Factor intercorrelations			O2.2		O3.2	O3.3		O4.2	O4.3	O4.4
		O2.1	0.51	O3.1	0.36	0.45	O4.1	0.37	0.30	0.47
				O3.2		0.37	O4.2		0.43	0.37
							O4.3			0.47

FUPC, first unrotated principal component; O4.1, class clown role; O4.2, comic talent; O4.3, disruptive rule-breaker; O4.4, subversive joker.

Table 1 shows that the first unrotated factor loaded on all items, and the coefficients range from 0.51 to 0.74 (median = 0.65). The highest loadings were by items with a high content saturation (“My classmates would call me a class clown,” “My classmates expect from me that I do silly things and make them laugh,” “Even when the teacher says my humor is disruptive, most of the time I cannot stop it immediately and need to continue having fun,” “During class it does not take long until my humor draws all attention of all my classmates on me”). The scores ranged from 1.00 to 5.56 (*M* = 2.54; Mdn = 2.44; *SD* = 0.85) with the majority of scores between 1 and 3 (i.e., “tend to disagree”). The mode was 2.22 (“disagree”) with only few high scorers. Nevertheless, skewness was non-significant (*Sk* = 0.51). There was no bimodality in the distribution of scores and no visible heap of class clowns at the upper end of the distribution. There were 13.1% of the children higher than 3.5 (the scale midpoint) and 5.4% were higher than 4.0 (“tend to agree to the behaviors”).

**Figure 1:**
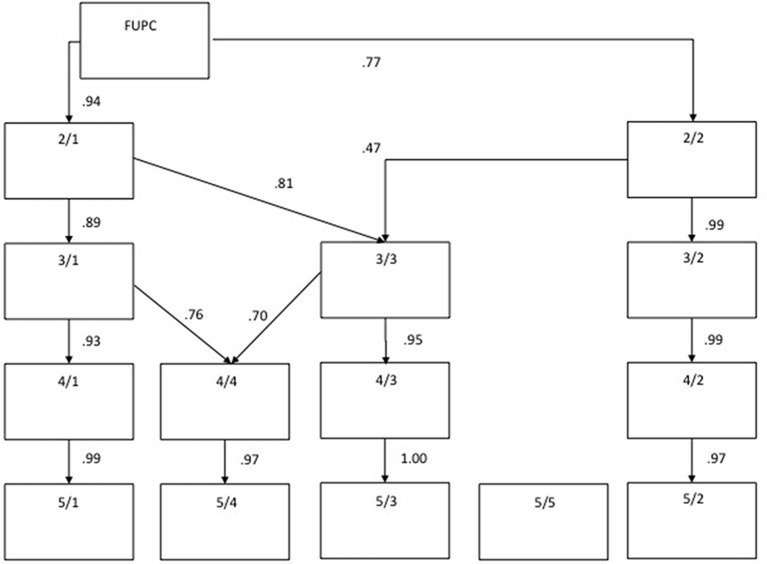
**Hierarchical factor analysis of the set of class clown items**.

The four-factor structure could be meaningfully interpreted and also the unfolding of the factors is telling (see Figure 1). The first Oblimin rotated factor was loaded by four items and was interpreted as “Identified as the class clown” as the salient loadings refer to having adopted a class clown role in class (α = 0.87). In addition to the two items forming the class clown index also the statements “My classmates expect from me that I do silly things and make them laugh” and “During class it does not take long until my humor draws all attention of all my classmates on me” loaded on this factor and made it clear that the high scorer was identified as the class clown. The second factor was loaded by five items and referred to a “Comic talent” (α = 0.83). Individuals with high scores described that they are quick with coming up with something funny. The hierarchical analysis showed that this factor was already there at step two and it did not change afterwards. The third factor was loaded by five items and referred to the “Disruptive rule-breaker” (α = 0.82). These individuals poke fun at things the teacher says, disregard rules and laugh at them. This factor emerged at step three (breaking away from the prior first one) and stays stable thereafter. The fourth factor (“Subversive joker”) referred to individuals that undermine the teachers authority (partly in their absence), competitively play pranks on others and only stop when having had made everyone laugh (α = 0.80). The fourth factor emerged at step 4, breaking away from the prior first one and also getting some variables from factor two^2^.

Table 1 also shows that the inter-correlation among the factors get gradually lower without containing any further pattern. In fact, a PCA of the four factors yielded a general factor that correlated with the FUPC to the extent of *r* = 0.99. It is noteworthy that the items of factor 1 also had the highest loadings on the FUPC. The second factor was normally distributed, but the others tended to be skewed (*Sk* = 0.84 − 0.94) with most scores at the lower end of the scale.

To examine whether class clowns and non-class clowns only differed in factor 1 but not the others the distinction into class clown and non-class clowns was correlated with the total score (minus the two items that form the index), factor 1 (composed of the remaining 2 items), and the three other class clown scales keeping gender and age constant. The correlations were high and significant, clearly demonstrating the class clowns yield higher scores overall (*r* = 0.47), identified as class clown (*r* = 0.55), the comic talent (*r* = 0.39), the disruptive rule-breaker (*r* = 0.36), and the subversive joker (*r* = 0.39). Thus, the class clowns (as a categorical distinction) are higher in all four dimensions of class clown behavior.

### The strengths of class clowns

Next, the first unrotated principal component and the four class clown factors were correlated with demographic variables, the five strengths factors and the 24 individual strengths. As the class clown data were correlated with gender and age, the latter correlations were controlled for age and gender. The results are displayed in Table 2.

**Table 2:** **Correlations between class clown dimensions and demographic variables, character strengths, life satisfaction and orientations to happiness**.

	**FUPC**	**O4.1**	**O4.2**	**O4.3**	**O4.4**
Age	0.07	−0.10^*^	0.29^*^	0.06	−0.09
Gender	−0.24^*^	−0.19^*^	−0.09	−0.21^*^	−0.23^*^
Leadership strengths	0.20^*^	0.17^*^	0.37^*^	0.00	0.02
Temperance strengths	−0.32^*^	−0.24^*^	−0.18^*^	−0.31^*^	−0.23^*^
Intellectual strengths	−0.04	0.01	0.07	−0.17^*^	−0.03
Transcendence strengths	−0.04	0.05	−0.06	−0.08	−0.04
Other-directed strengths	−0.17^*^	−0.08	−0.06	−0.19^*^	−0.18^*^
Creativity	0.00	0.02	0.12^*^	−0.16^*^	0.01
Curiosity	−0.02	0.00	0.09	−0.10	−0.05
Open-Mindedness	−0.08	−0.06	0.03	−0.16^*^	−0.08
Love of learning	−0.17^*^	−0.09	−0.07	−0.26^*^	−0.11^*^
Perspective	0.07	0.07	0.23^*^	−0.09	−0.01
Bravery	0.04	0.08	0.15^*^	−0.07	−0.06
Perseverance	−0.19^*^	−0.07	−0.07	−0.26^*^	−0.15^*^
Honesty	−0.24^*^	−0.11^*^	−0.10	−0.27^*^	−0.23^*^
Zest	0.04	0.09	0.17^*^	−0.13^*^	−0.05
Love	0.01	0.04	0.13^*^	−0.10	−0.07
Kindness	−0.04	0.03	0.04	−0.10	−0.10
Social Intelligence	−0.03	−0.03	0.15^*^	−0.15^*^	−0.10
Teamwork	−0.08	−0.04	0.08	−0.19^*^	−0.12^*^
Fairness	−0.14^*^	−0.08	−0.04	−0.17^*^	−0.15^*^
Leadership	0.14^*^	0.11^*^	0.24^*^	0.00	0.06
Forgiveness	−0.02	0.03	0.05	−0.10^*^	−0.06
Modesty	−0.18^*^	−0.14^*^	−0.10	−0.15^*^	−0.15^*^
Prudence	−0.25^*^	−0.17^*^	−0.13^*^	−0.26^*^	−0.19^*^
Self-regulation	−0.23^*^	−0.19^*^	−0.10^*^	−0.22^*^	−0.18^*^
Appreciation of beauty and excellence	−0.03	0.04	0.03	−0.15^*^	−0.02
Gratitude	−0.02	0.02	0.09	−0.09	−0.08
Hope	−0.01	−0.03	0.13^*^	−0.07	−0.09
Humor	0.37^*^	0.34^*^	0.51^*^	0.10^*^	0.11^*^
Religiousness	−0.02	0.04	−0.08	−0.03	0.03

N = 630. FUPC, first unrotated principal component; O4.1, class clown role (Identified as the class clown); O4.2, comic talent; O4.3, disruptive rule-breaker; O4.4, subversive joker.

* p < 0.01.

Table 2 shows that males were higher than females in all clown dimensions except for the second, the “Comic talent,” which is not gender specific. The younger participants tended to identify as the class clown more often than the older while the comic talents were more prevalent among the older. The correlations with the strengths factors gave a clear pattern of results. All class clown dimensions were low in temperance (confirming Hypothesis 1), the two negative ones (and the FUPC) were low in other-directed strengths (Hypothesis 4) and the factors 1 and 2 (and the FUPC) were high in leadership (Hypothesis 3). The “Disruptive rule breaker” was additionally lower in intellectual strengths (Hypothesis 5). The correlations with the 24 character strengths were examined next and the overall class clown dimension and the four types yielded different correlational profiles. The FUPC was positively related to humor and leadership, and negatively with seven strengths including prudence, self-regulation and honesty. The identification with the class clown role went along with humor and leadership and lower scores in the strengths of prudence, modesty, self-regulation and honesty. Thus, overall the correlation pattern was similar to the one of the FUPC. The “Comic talent” was characterized by nine strengths (most notably humor, leadership, and perspective) and by the absence of two strengths (prudence and religiousness). The “Disruptive rule breaker” was slightly higher in humor but lower in 14 of the 24 strengths (most noticeably, in honesty, prudence, love of learning, and perseverance). The “Subversive joker” tended to be slightly higher in humor, but also lower in eight strengths, but these correlations were low except the one with honesty.

### Signature strengths of class clowns

**Figure 2:**
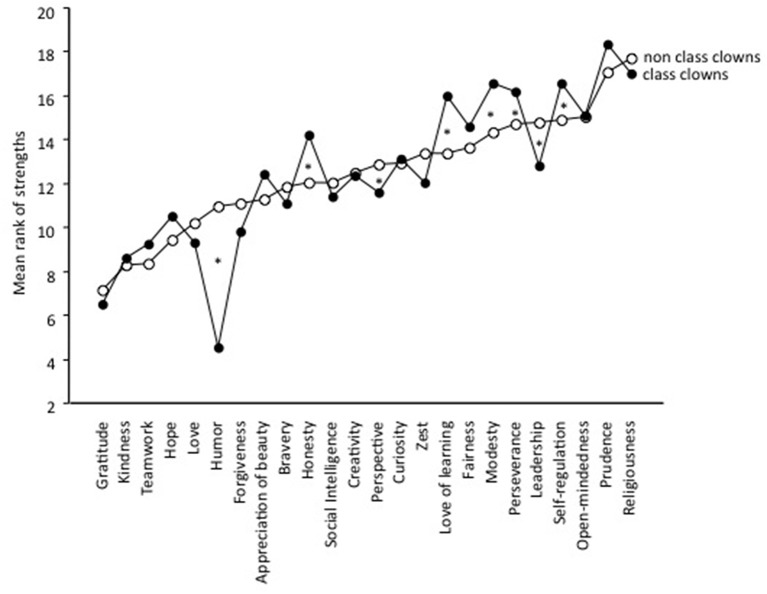
**Mean ranks in the 24 strengths for class clowns and non-class clowns separately**.

A final analysis looked at the rank of the character strengths of class clowns and non-class clowns. This analysis disregards the level of strengths but looks at the order of strengths for each individual. For each student the 24 YIA-Youth scores were ranked (from 1 to 24) and the mean profiles for class clowns and non-class clowns were computed and is displayed in Figure 2.

For class clowns, the ranks were higher for humor (*r* = 0.29; *p* < 0.01), leadership (*r* = 0.09; *p* < 0.01) and perspective (*r* = 0.08; *p* < 0.05), and lower for love of learning (*r* = −0.13; *p* < 0.01), honesty (*r* = −0.12; *p* < 0.01), modesty (*r* = −0.11; *p* < 0.01), self-regulation (*r* = −0.09; *p* < 0.05), and perseverance (*r* = −0.08; *p* < 0.05). While humor seemed to be a signature strength of class clowns (mean rank = 4.5; compared to the mean rank of non-class clowns: 11.0), perspective (mean rank: 11.6) and leadership (mean rank: 12.8) do not show up among the top strengths although it does more frequently than for non-class clowns. The lowest ranked strengths are love of learning (*M* = 16.0), perseverance (*M* = 16.2), self-regulation (*M* = 16.6), modesty (*M* = 16.6), religiousness (*M* = 17.0), and prudence (*M* = 18.4). It should be noted that while these were the lowest ranked strengths among the class clowns the mean ranks do not reach as far to the end of the scale as humor does. The fact that humor is a signature strength of class clowns was underscored by the fact that 29.1% had it place as the top strength (compared to 7.7% of the non-class clowns), 62.8% among the top three (non-class clowns = 20.7%), and 75.5% (non-class clowns = 30.1%) among the top five strengths. This confirms Hypothesis 7 (the numbers for the subfactors were 81.1, 55.4, 61.9, and 51.6%, respectively). The fewest strengths class clowns had among their highest five were prudence: 1.2% (non-class clowns: 5.9%), modesty: 4.8% (non-class clowns: 14.2%), and self-regulation: 7.1% (non-class clowns: 8.9%).

Finally, we examined humor as a signature strength balanced and unbalanced by prudence. Individuals with humor among the top five strengths were separated from those that did not have humor as signature strength. Furthermore, a second distinction was made depending on prudence was among the top 12 strengths or lower 12 strengths^3^. This allows testing the effects of prudence or low prudence among people for whom humor is a signature strength (i.e., Hypothesis 8). A 2 × 2 ANCOVA was performed with humor and prudence as independent variables and the rule breaker scores as a dependent variable (and gender and age as covariates).

**Figure 3:**
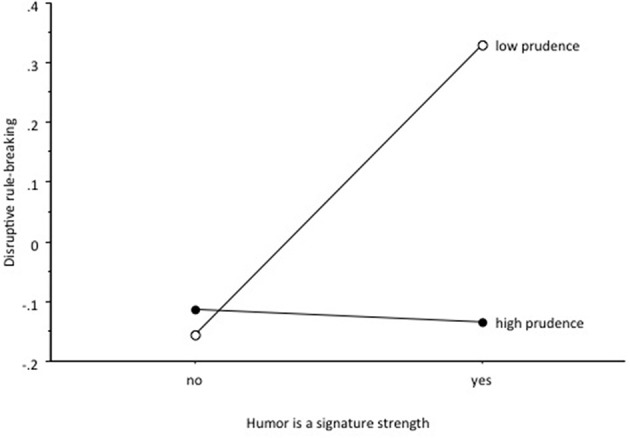
**Interactive effects of humor as a signature strengths (yes, no) and prudence (high, low) on the degree of disruptive rule-breaking**.

There was a significant interaction between humor and prudence, *F*_(1, 627)_ = 5.27, *p* < 0.05, η^2^_*p*_ = 0.008. Only the combination of humor as a signature strength and low expression of prudence went along with high scores in being a disruptive rule breaker (Figure 3). If prudence was high, it did not matter whether humor was the signature strength or not. When prudence was low then humor was a condition for disruptive rule-breaking.

### The class clowns orientations to happiness and satisfaction with life

Finally, the class clown data were correlated with the orientations to happiness (OTH) and life satisfaction (Hypothesis 9). Table 3 shows the correlations.

**Table 3:** **Correlation between class clown dimensions and orientations to happiness and global satisfaction with life controlled for age and gender**.

	**FUPC**	**O4.1**	**O4.2**	**O4.3**	**O4.4**
Pleasure (OTH)	0.29^*^	0.25^*^	0.26^*^	0.18^*^	0.17^*^
Engagement (OTH)	−0.09	0.08	−0.04	−0.23^*^	−0.08
Meaning (OTH)	0.02	0.13^*^	0.03	−0.06	−0.04
SLSS	−0.05	−0.02	0.04	−0.12^*^	−0.06

N = 338. FUPC, first unrotated principal component; O4.1, class clown role; O4.2, comic talent; O4.3, disruptive rule-breaker; O4.4, subversive joker.

* p < 0.05.

From Table 3 it is clear that class clown behavior did go along with a life of pleasure; i.e., class clowns follow the principle of hedonism and maximize pleasure (Hypothesis 2). There was a small positive correlation between life of meaning and “identifying as the class clown.” The “disruptive rule-breaker” was lower in life of engagement; i.e., they do not get immersed in challenges, don't experience flow but rather the opposite (supporting Hypothesis 6). More importantly, there was also a lower correlation with global life satisfaction (confirming Hypothesis 9). One should add that humor and life satisfaction were positively correlated, *r* = 0.23, *p* < 0.001 (controlled for gender and age). Removing humor (and age and gender) from the relationship between class clowning and life satisfaction yielded negative correlations for the FUPC (*r* = −0.12, *p* < 0.01), the factors “Identified as a class clown” (*r* = −0.12, *p* < 0.01), “Disruptive rule breaker” (*r* = −0.10, *p* < 0.01), and “Subversive joker” (*r* = −0.08, *p* < 0.05), but not the “Comic talent” (*r* = −0.06, *p* = 0.13). Finally, partial correlations show that removing humor (in addition to age and gender) does not substantially reduce the correlation between the FUPC and the life of pleasure (*r* = 0.22; *p* < 0.001); i.e., the liking of pleasure of class clowns goes beyond the effects of humor. However, the overall class clown behavior (i.e., the FUPC) is now significantly negatively correlated with life of engagement (*r* = −0.18; *p* < 0.001), while the negative correlation with life of meaning fails to be significant (*r* = −0.09; *p* = 0.09).

## Discussion

The present study allows three major conclusions. First, it is evident that a behavior-centered, dimensional approach to class clowning is possible if not favorable. Second, class clown behavior can be described in this dimensional approach using a hierarchical model with a broader factor on the top that unites four positively related lower order dimensions. Third, variables from positive psychology (e.g., character strengths, orientations to happiness) are well-suited to predict why certain people are involved in class clowning and others aren't. They help drawing an overall picture of class clowns and contribute to the differentiation of the four dimensions.

Indeed, the dimensional self-report approach proposed in the present article can be seen as a valuable complement to the person-centered type approach based on teacher or peer nominations as it replicates insights from the latter but goes beyond it. Also in the present study the different estimations of the frequency of class clowns assume the number to be low and vary around 10% (teachers nomination: 11%, self-report: 13%, total score > 3.5: 13%, total score > 4: 5%). Furthermore, both the teachers nominations and the self-reports (items 4 and 9, and the total score) showed that there is variations among the class clowns: they were nominated by a varying number of teachers (13–83%) and the magnitude of agreement to the class clown questions varied, too (between 4 and 5.6 on the 6 point scale; i.e., between “slightly agree” and “strongly agree”). Thus, the agreement is far from being perfect. Moreover, in the distribution of the continuous data no apparent discontinuity could be observed and the distribution was not bimodal. Thus, while it is possible to divide students into class clowns and non-class clowns, this distinction will remain arbitrary. The outcome depends on the measurement used, the boundaries are blurry and do not allow for a clear cut-off score, and there is a lot of variance within both groups. Again, these factors speak for a dimensional approach, which also approximates the common observation that the actual high scorers are rare and most people do not involve in class clown behavior. Finally, also in the dimensional approach boys seem to be the gender involved in class clowning; while only 38% of the sample were boys, they were 56% of the class clowns. Should the dimensional approach be applied in the future more research is needed on defining and justifying cut-off scores.

The second major outcome is a tentative descriptive model along with a preliminary instrument for its assessment. We propose to study class clown behavior at two levels. At the first level, the data are well represented by a strong general factor (i.e., the first unrotated principal component) loaded highly by all items reflecting the amount someone is involving in class clown behavior. The sum of the 18 items yields a reliable measure that also is reasonably balanced across the four domains. Therefore, the total score is recommended for use in studies. At the second level of analysis, this general factor may be split up into four correlated components, namely the “Identified as a class clown,” “Comic talent,” “Disruptive rule breaker” and the “Subversive joker.” Factor 1 (i.e., being identified as a class clown) describes that pupils have adopted the role of a class clown. While factors 2 to 4 describe different styles of class clown behaviors, this factor represents the crystallization of showing these behaviors for a while (Hobday-Kusch and McVittie, 2002). The high scorer had used humor to negotiate power with teachers and gaining approval or at least attention from their peers. This also includes that the class clown is aware that others expect certain actions from them. We expect that the scores get higher with time. Factor 2 (“the comic talent”) refers to a class clown behavior that is based on quick-wittedness and is more characterized by spreading good cheer and entertaining others. This type of class clown behavior might be seen as less disturbing by the teachers and it might even be the humor that is welcome also in schools. More people did show it (compared to the other factors) and the scores were normally distributed. It is likely that this class clown is not only liked by their peers but also accepted by teachers. He or she will have a certain status in class, maybe being the second leader in class after the teacher. The other two class clowning dimensions are more conflict-prone as they go against classroom rules and challenge the teacher. The “disruptive rule breaker” is the visible opponent of the teacher; he or she does not take seriously what the teachers say, dismisses what is said to be important, pokes fun what that the teacher says or does and undermines his authority. The “subversive joker” is undermining the authority of the teacher but not necessarily in direct confrontation. He or she also plays pranks on classmates. He is competitive in playing pranks and needs the attention of the class. These labels are preliminary and might be updated once a new batch of items will be studied together with the present item pool. For now, the factor scores serve as preliminary measures of the dimensions but a further development of the instrument should have subscales.

As a third major outcome one can state that the concepts of character strengths, signature strengths and orientations to happiness are useful in predicting individual differences in class clown behavior and allowed to test several hypotheses regarding overall class clowning, as well as the four dimensions. Overall, class clown behaviors are more frequent among those lower in temperance strengths (in particular prudence) (Hypothesis 1), but high in life of pleasure and humor (Hypothesis 2). Indeed, humor is the only common signature strengths among class clowns (Hypothesis 7), and it may be conducive to be applied in a less appropriate place especially if it is not balanced out by prudence (Hypothesis 8). These dispositions are predictive of all forms of class clowns and they might be relevant at different stages in class clown behaviors. An orientation toward pleasure might extend to the classroom situation that is not conducive to having fun. Humor is a vehicle for producing amusement, which, if someone lacks prudence/temperance, also gets expressed in a setting where it might not be appropriate or even gets sanctioned. In the study by Damico and Purkey (1978) class clowns were found to be high in “cheerfulness,” but this was a smaller effect. This might be due to the fact that cheerfulness and humor only overlap partially.

Specific predictors of the four dimensions complement this nucleus of class clown dispositions. Damico and Purkey (1978) found class clowns to be high in leadership (Hypothesis 3). The present study shows that this applies primarily to the “Comic talent,” but can also be found for the “Identified as a class clown”-dimension and the first unrotated factor. While the two negative dimensions of class-clown behaviors (i.e., “rule breaking” and “subversive joking”) do not go along with leadership they are typical for students low in other-directed strengths (Hypothesis 4). Playing pranks and breaking rules in classroom is more likely among those with lower orientations to the community. It should be noted that the scores in “comic talent” and “identified as a class clown” and do not predict undermining teachers authority or breaking the rules set. Furthermore, it seems that intellectual strengths and pursuing a life of engagement are protective factors against disruptive rule breaking (Hypothesis 5, Hypothesis 6). Being equipped with school-related strengths (e.g., love of learning, perseverance) and disposed toward flow will leave little room for being distracted and bored at school, which might be one base of triggering class clown behavior. Finally, life satisfaction tended to correlate negatively with all class clown dimensions (Hypothesis 9). This was significant for the disruptive rule breakers (zero-order correlations) and all dimensions except the comic talent (partial correlations controlling for age, gender and humor). This finding is interesting, as humor as a strength may generate positive emotions and improve social relations. Hence humor is typically predictive of life satisfaction and well-being (Ruch et al., 2010). However, humor in an inappropriate setting may also lead to negative consequences, and thus overall detrimental to well-being. Future studies should examine the relationship with well-being at school or satisfaction with school experiences, as these will reflect the class clown effects more strongly and mediate the relationship between humor in schools and global life satisfaction. Overall the findings for the four factors suggest that it is fruitful to distinguish dimensions of class clown behaviors and stop looking at it as a unitary concept.

These initial findings need to be interpreted in the context of some limitations. A prime limitation is the snapshot characteristic of the results. It does not take into account that strengths develop or change as a function of experience. The results are interpreted as the strengths facilitating the class clowning behavior. Right now we see that, for example, the rule breaker is lower in love of learning, endurance, honesty, and prudence and one might speculate that lower expressions in these strengths facilitate class clown behavior. The causality could be in the other direction, being the humorous opponent of the teacher might also shape the leadership role. And it might be that other variables affect both the emergence of class clown behaviors and character. Also, the current approach does not look for interactions with type of subject taught and with teacher's characteristics. We do not know whether class clowning is depending on these factors or independent from them. Another limitation is that the present study takes all age ranges together. It might be that the nature of the clowning behavior changes with age (e.g., from behavioral to verbal humor, or from doing silly things to doing targeted attacks). It is also possible that the type of school will play a moderating role. Is it a class of academically gifted students, or a class where pupils are prepared to work in a vocational profession?

The low correlation with the humor scale requires some further discussion. While humor is the only consistent signature strength of class clowns it is also obvious that mostly the “Comic talent” dimension is predicted by the VIA-Youth humor scale. The correlations with the “Disruptive rule-breaker” and “Subversive joker” dimensions are rather low. This might be explained by the fact that in the VIA-Classification of Strengths humor is intentionally restricted to forms of humor that serve some moral good. Peterson and Seligman (2004) did define the humorous individual as one “… who is skilled at laughing and teasing, at bringing smiles to the faces of others, at seeing the light side, and at making (not necessarily telling) jokes (p. 530).” Empirical studies using the VIA-IS scale (and other conceptualizations of humor) show that humor is most strongly related to humanity. However, subversive and disruptive class clowning will not be guided by humanity; i.e., it is amusing others by tricks, jokes, odd gestures and postures, or pranks. Seligman (2014) discusses that in Petersons model of the “real mental illnesses” the excess of humor as a strengths might be buffoonery; i.e., “ridiculous but amusing,” foolish or playful behavior or practice. *This “excess of humor” is not measured through the VIA-IS.* Research in adults has shown that humor is multidimensional involving also more negative forms of humor, such as mean-spirited or earthy humor. Interestingly, Müller and Ruch (2011) demonstrated that earthy or mean-spirited types of humor were negatively related to temperance; i.e., modest, prudent or self-regulated individuals less often indulged in them. Thus, future studies should use other components of humor to predict the class clown behavior. Katagelasticism (i.e., the joy of laughing at others) and other forms or corrective humor (Ruch and Proyer, 2009) might be more characteristic for the disruptive rule breaker and the subversive joker while at the same time being less predictive of the comic talent. Likewise, a more comprehensive approach could be used (e.g., the HBQD by Craik et al., 1996). In such a study the systematic comparison of teacher evaluations with self-reports and peer-reports describing the behavior are of interest as Damico and Purkey (1978) reported that some teachers fail to distinguish between comic and hostile humor, and classify it all as disruptive without seeing positive ways in which to use humor to meet their own objectives. Furthermore, a study is needed that collects examples of humor shown in class and actually analyses the type of humor displayed. It is possible that some of the behaviors shown are merely disruptive or rude and do not contain elements of humor. Alternatively and more likely, the behaviors are better described as ridicule, laughing at, sheer mockery, parody, or corrective humor. They might also be more imaginative. Clearly, studies are needed that analyze the nature of the funny behavior (if it is funny at all).

## Conclusions

The “class clown” is better conceptualized as continuous rather than categorical and as multi- rather than unidimensional. Humor is the shared signature strength of 75% of the class clowns. Furthermore, the class clowns do have strengths, and one of the dimensions goes along with even several strengths. The apparently more antagonistic class clown factors seem to be associated with lower expressions in strengths. The rule breaker is lower in life of engagement. Thus, activating the strengths that are conducive to positive affect might be a way to indirectly approach the problems (Weber and Ruch, 2012; Weber et al., submitted). However, the dynamics must be better understood. For teachers there will be the challenge of preventing class clown behavior by considering the conditions that bring about this behavior and to mastering class clown behavior more effectively. Maybe the concept of use of signature strengths will be important in this context. In general humor serves a variety of functions (e.g., it manages relationships, it buffers stress, it energizes, it helps influencing) and some of these are highly relevant at school. The teacher might use humor to melt down conflicts and tension with humorous remarks, highlight a point with humor so that it is more easily remembered, or humor can make students laugh and be distracted but then alert again after laughter etc. Students with humor as a signature strengths will want to use humor too during class or during breaks. When humor interrupts the flow of teaching, or is directed at classmates or the teacher it can be seen as a misuse of a strength (Webb, 1994). When it is used constructively students might use it for building relations, leading, influencing or highlighting points, energizing, resolving conflicts, managing emotions etc. Generally the school setting will favor strengths like perseverance, love of learning, prudence, self-regulation, teamwork and social intelligence (Weber and Ruch, 2012; Weber et al., submitted) and students having these strengths among their signature strengths will thrive in this context more easily than those who don't. Naturally, the fit between humor and a teaching institution might me lower at first glance, but if humor is granted a place in school it will help students with humor as a signature strength to feel at home at school as well. Whether or not they don't use humor in a detrimental way needs to be studied.

### Conflict of interest statement

The authors declare that the research was conducted in the absence of any commercial or financial relationships that could be construed as a potential conflict of interest.

## Footnotes

^1^Separate factor analyses were also computed for the individuals indicating to be class clowns (>3.5) and those who don't. For both samples there were clearly 4 factors (Eigenvalues for the class clown group: 5.73, 2.23, 1.62, 1.51, 0.96, and 0.89).

^2^Separate factor analyses were computed for boys and girls and the factor structures turned out to be highly similar. An analysis of the 158 primary schoolers separate from the secondary schoolers yielded a less clear structure for the primary schoolers. Mostly the subversive joker factor is not yet there.

^3^The choice of the “top five” for humor is in line with the idea that there are “signature strengths” and that their number is assumed to be between 3 and 7. In most studies five signature strengths are assumed. Prudence is not a signature strength, so we somewhat arbitrarily divided individuals into “high” and “low” using the numerical midpoint (i.e., 12.5).
